# Round the Hospitals

**Published:** 1916-07-29

**Authors:** 


### ROUND THE HOSPITALS.

By the time the present issue of The Hospital is in the hands of the majority of our readers we hope an agreed Bill providing for the registration of certificated trained nurses and establishing the College of Nursing, with statutory powers, will have assumed its final form. If it is safe to rest an opinion on the statements contained in certain articles appearing in some of our nursing contemporaries last week, it is clear every efficient and knowledgeable trained nurse will for her own protection support the demand that the Bill shall contain the names of the forty-five members who are to constitute the Council of the College for the next two years and to have charge of the Register. It will not be an easy task to name and appoint these forty-five persons, who will have great responsibility and the majority of whom should be known and trusted workers in nursing affairs.

The first two years will be years of crisis and difficulty, far in excess of any which are likely to follow them, and no Bill can be made acceptable to the nursing profession as a whole at the present time unless it contains the names of the Council which will exercise control during the intermediate period represented by the first two years after the Bill has passed through Parliament and received the Royal assent. Every independent person and every wise nurse demands the inclusion of the names in the Bill as the surest guarantee that they will be representative and trustworthy in the highest meaning of these words. There may be individuals with axes to grind or ends to serve who may favour the omission of the names of the first Council. If any such there be, they should be stoutly resisted by every independent and intelligent member of the nursing profession. Even the withdrawal of a few individuals at the last moment would be a matter of small importance compared with the defeat of the policy to suppress all names and keep the nurses in ignorance of the actual people in whose hands will rest the destiny of the College of Nursing and the Register of Trained Nurses during the first two years succeeding their creation with statutory powers.

The London Hospital and all connected with it are full of satisfaction at the arrangement which has been made to provide accommodation there for wounded officers. It is a long, long way to Whitechapel, but when the officers get there they will find good reason to be pleased and satisfied at their reception and treatment. It would be difficult to find anything which could have given greater pleasure to the authorities, and especially to the matron, sisters, and nurses at the London Hospital.

We have given in recent numbers (see The Hospital, February 19, 1916, p. 459; May 13, 1916, p. 156) some interesting articles on the Incorporated Society of Trained Masseuses, its examinations and objects. Since our last issue it has been authoritatively announced that the course of instruction for candidates for the examinations would shortly be extended from six months to one year, such course to include full instruction in Swedish remedial exercises. Matrons, sisters, and nurses are anxious to ascertain exactly what are the bearings of this new departure. Will the new regulations include a shorter course for the instruction of certificated trained nurses, or does the Incorporated Society wish to deter them from becoming fully qualified masseuses, seeing that trained nurses, with but very few exceptions, may find it impossible to devote a fifth year to training in massage and Swedish remedial exercises? We favour the institution of a shorter course for the trained nurse, seeing the large number of nurses in private work who need this knowledge, and it is not generally possible for a nurse-training school as at present organised to arrange to include massage in the fourth year's curriculum. We advise practitioners and teachers, matrons and sisters, to look into this matter, and shall be glad to have correspondence expressing the opinion generally held in the important matters mentioned. We think it desirable for the Incorporated Society to delay the commencement of the year's course system until after the termination of the war.

The silver badge which, on the recommendation of the Army Council, the King has approved to be issued to officers and men of the British, Indian, and Oversea Forces who have served at home or abroad since August 4, 1914, and who have retired or been discharged from the Army owing to physical infirmity arising from wounds or sickness caused by military service, or on account of age, will also be awarded under the same conditions to members of Queen Alexandra's Imperial Military Nursing Service, Regular, Reserve and Territorial Force, Queen Alexandra's Nursing Service for India, and members of Voluntary Aid detachments. No mention is made of the nurses serving under the Joint War Committee, the Civil Hospitals Reserve, or the Nursing Service of the various Colonies. The Admiralty also announce that a similar badge will be issued to members of Queen Alexandra's Royal Naval Nursing Service under parallel conditions. Full instructions will be published later, stating when and to whom applications should be addressed by persons entitled to these badges. The warning is added that *Premature applications will only cause delay.*

On her retirement as a sister of the Women's Medical Ward of the South Devon and Exeter Hospital, Miss M. L. Salmon has been presented by Miss Hopkins with a gold wrist-watch from the matron, sisters, and nurses. Miss Hopkins expressed her appreciation of the good work performed by Sister Salmon for so many years, and wished her every happiness and benefit in the rest she had so well earned. Sister Salmon, who has been attached to the staff for over twenty years, had endeared herself to her colleagues as a loyal and reliable friend, whilst her kindness to her patients was well known.

**For Matrons' and Sisters' Appointments see p. iv, and The Control of Lay Women in Hospitals see p. 416.**

